# Six-transmembrane epithelial antigen of the prostate and enhancer of zeste homolog 2 as immunotherapeutic targets for lung cancer

**DOI:** 10.1186/1479-5876-9-191

**Published:** 2011-11-05

**Authors:** Satoshi Hayashi, Takumi Kumai, Yoshiya Matsuda, Naoko Aoki, Keisuke Sato, Shoji Kimura, Masahiro Kitada, Masatoshi Tateno, Esteban Celis, Hiroya Kobayashi

**Affiliations:** 1 Department of Surgery, Asahikawa Medical University, Asahikawa, Japan; 2 Department of Pathology, Asahikawa Medical University Asahikawa, Japan; 3 H. Lee Moffitt Cancer Center and Research Institute, Tampa, FL, USA

## Abstract

**Background:**

T-cell based immunotherapy for lung cancer (LC) could be a promising and novel therapeutic approach. Six-transmembrane epithelial antigen of the prostate (STEAP) and the polycomb group protein enhancer of zeste homolog 2 (EZH2) are highly expressed in LC and since the expression of molecules in normal tissue is significantly lower as compared to tumor cells, these proteins are considered as potential tumor-associated antigens (TAAs) for developing T-cell based immunotherapy.

**Methods:**

We assessed the capacity of predicted CD4 T-cell epitopes from STEAP and EZH2 to induce anti-tumor immune responses to LC cell lines.

**Results:**

Out of several predicted epitopes, two synthetic peptides, STEAP_281-296 _and EZH2_95-109_, were effective in inducing CD4 T-cell responses that were restricted by HLA-DR1, DR15, or DR53 molecules, indicating that the peptides function as promiscuous T-cell epitopes. Moreover, STEAP_281-296 _and EZH2_95-109_-reactive T-cells could directly recognize STEAP or EZH2 expressing LC cells in an HLA-DR restricted manner. In addition, some STEAP-reactive T-cells responded to STEAP+ tumor cell lysates presented by autologous dendric cells. Most significantly, both of these peptides were capable of stimulating *in vitro *T-cell responses in patients with LC.

**Conclusions:**

Peptides STEAP_281-296 _and EZH2_95-109 _function as strong CD4 T-cell epitopes that can elicit effective anti-tumor T-cell responses against STEAP or EZH2 expressing LC. These observations may facilitate the translation of T-cell based immunotherapy into the clinic for the treatment of LC.

## Background

Lung cancer (LC) represents a significant health problem with 222,520 new cases and 157,300 deaths in the past 10 years in the United States [1]. Recently, adjuvant cisplatin-vinorelbine chemotherapy in completely resected non-small cell LC (NSCLC) has resulted in an enhanced survival benefit at 5 years (8.9% improvement versus observation) [2]. Molecular target-based drugs (gefitinib, erlotinib, etc.) are available for specific types of NSCLC showing epidermal growth factor receptor mutations. However, these chemotherapeutic regimens can be extremely toxic and provide limited survival benefit for advanced LC. Thus, the development of novel and less toxic alternatives such as immunotherapy is warranted. Nevertheless, the success of immunotherapy will ultimately rely on the identification of appropriate tumor-associated antigens (TAAs) that are overexpressed in tumor cells relative to normal tissues.

Six-transmembrane epithelial antigen of the prostate (STEAP) is a 339 amino acid protein that is critical for erythroid iron homeostasis. STEAP is located on the cell surface [3] and is predominantly overexpressed in various tumor types (prostate, bladder, colon, ovarian, and Ewing sarcoma) [4]. The enhancer of zeste homolog 2 (EZH2) is a polycomb group protein that functions as a regulator of homeobox gene expression [5]. EZH2 is highly expressed in various tumor types including prostate [6], breast [7], esophagus [8], and pancreatic [9] cancers. Moreover, the expression of EZH2 has been linked to tumor aggressiveness and metastatic potential, and has been linked to a poor overall patient prognosis [6-8]. The low expression of STEAP and EZH2 in normal tissues together with recent studies reporting that these molecules are overexpressed in NSCLC, suggests that both protein could be utilized as TAAs in LC [10,11].

Although CD8 cytotoxic T lymphocytes are believed to have a major role in eradicating cancer, CD4 helper T lymphocytes are likely to have a critical role in immunotherapy since they participate in generation and persistence of CD8 T-cell responses [12]. In addition, CD4 T-cells exhibit an effector role against tumors that express HLA-DR molecules [13]. For the development of peptide-based immunotherapies against LC, we have searched for possible HLA-DR epitopes capable of eliciting CD4 T-cell responses to STEAP and EZH2. Here, we report that 2 epitopes, STEAP_281-296 _and EZH2_95-109 _were capable of eliciting *in vitro *antigen-specific, HLA-DR-restricted CD4 T-cell responses against LC cells expressing STEAP and EZH2. In addition, peptides STEAP_281-296 _and EZH2_95-109 _were also found to stimulate T-cell responses in LC patients. We believe that these results may be of use for the development of T-cell based immunotherapy for LC.

## Methods

### Cell lines

Mouse fibroblast cell lines (L-cells) transfected and expressing individual human HLA-DR molecules were kindly provided by Dr. Robert W. Karr (Karr Pharma, Saint. Louis, MO, USA) and by Dr. Takehiko Sasazuki (Kyushu University, Fukuoka, Japan). The LC cell lines PC14, A549, LC-2/ad, LCAM1 (adenocarcinomas), LK2, RERF-LC-AI, EBC1 (squamous cell carcinomas), LU65, and LU99 (large cell carcinomas) were supplied by the RIKEN Bio-Resource Center (Tsukuba, Japan). The LC cell lines LHK2 and 1-87 (adenocarcinomas) were kindly provided by Dr. Yasuaki Tamura (Sapporo Medical University, Sapporo, Japan). Tumor cell lines H23, H441 (lung adenocarcinomas), H520, SK-MES-1, Calu-1 (lung squamous cell carcinomas), PC3 (prostate cancer), MCF7 (breast cancer), WiDr (colon carcinoma) and Jurkat (T cell lymphoma) were purchased from ATCC (Manassas, VA, USA). All cell lines were maintained in tissue culture as recommended by supplier.

### Immunohistochemistry

An indirect immunoperoxidase technique (the streptavidin-biotin method) was performed. To detect STEAP, polyclonal rabbit anti-human STEAP (ZMD.265, Zymed Laboratories, Inc., South San Francisco, CA, USA), diluted 1:200, served as the primary antibody. To detect EZH2, monoclonal mouse anti-human EZH2 (BD.612666, BD Bioscience, San Jose, CA, USA), diluted 1:200, served as the primary antibody. To detect HLA-DR, monoclonal mouse anti-human HLA-DR α chain (TAL.1B5, DakoCytomation, Denmark), diluted 1:10000, served as the primary antibody. We assessed STEAP, EZH2 and HLA-DR expression in surgically resected LC specimens.

### Western blotting

One million cells were washed in phosphate buffered saline (PBS) and lysed in NuPAGE LDS sample buffer (Invitrogen). The cell lysate was subjected to electrophoresis in a 4-12% NuPAGE bis-Tris SDS-PAGE gel (Invitrogen) under reducing condition and transferred to Immobilon-P (Millipore, Bedford, MA, USA) membrane. The membrane was blocked in PBS containing 0.01% Tween 20 and 5% non-fat dry milk for 1 h at room temperature and incubated with rabbit anti-human STEAP polyclonal antibody (H-105, Santa Cruz Biotechnology, Santa Cruz, CA, USA) diluted 1:200 in blocking buffer, anti-human EZH2 mouse monoclonal antibody (mAb) (BD.612666, BD Bioscience, San Jose, CA, USA) diluted 1:200, or anti-β-actin mAb (C4, Santa Cruz Biotechnology, Santa Cruz, CA, USA) diluted 1:1,000 in blocking buffer as the control, for 18 h at 4°C. After washing, the membrane was incubated with horseradish peroxidase-labeled sheep anti-rabbit or -mouse IgG and subjected to the enhanced chemiluminescence assay using the ECL detection system (Amersham, Buckinghamshire, UK).

### Synthetic peptides

Potential HLA-DR-restricted CD4 T-cell epitopes were selected from the amino acid sequence of STEAP and EZH2 using the computer-based algorithms peptide/MHC binding for three common HLA-DR alleles (DRB1*0101, DRB1*0401, DRB1*0701) developed by Southwood et al. [14]. The predicted peptide epitopes were synthesized by solid phase organic chemistry and purified by high-performance liquid chromatography (HPLC). The purity (> 80%) and identity of peptides were assessed by HPLC and mass spectrometry, respectively. The synthetic peptides used throughout this study were STEAP_261-275 _(SLLLGTIHALIFAWN), STEAP_281-296 _(KQFVWYTPPTFMIAVF), EZH2_95-109 _(VIPLKTLNAVASVPI), EZH2_220-234 _(SDKIFEAISSMFPDK), EZH2_693-704 _(PNCYAKVMMVNGDHR). These peptides were selected on the basis of having top 10 scores for at least 2 of the three HLA-DR alleles. The tetanus toxoid (TT) _830-843 _(QYIKANSKFIGITE) peptide was used as a control universal epitope peptide, since it is presented by multiple HLA-DR alleles [15].

### *In vitro *induction of antigen-specific CD4 T-cell clones with synthetic peptides

The procedure utilized for the generation of STEAP and EZH2 reactive CD4 T-cell clones using peptide-stimulated lymphocytes from peripheral blood mononuclear cells (PBMCs) of human healthy individuals has been described in detail [16]. Briefly, dendritic cells (DCs) were produced from purified CD14 monocytes (using antibody-coated magnetic microbeads from Miltenyi Biotech, Auburn CA, USA) cultured for 7 days at 37°C in a humidified CO_2 _(5%) incubator in the presence of 50 ng/ml GM-CSF and 1,000 IU/ml IL-4. Peptide-pulsed DCs (3 μg/ml for 2 h at room temperature) were irradiated (4,200 rad) and co-cultured with autologous purified CD4 T-cells in 96-flat-bottomed-well culture plates. One week after peptide stimulation, the CD4 T-cells were restimulated in individual microcultures with peptide-pulsed irradiated autologous PBMCs and 2 days later, recombinant human IL-2 was added at a final concentration of 10 IU/ml. One week later, the T-cells were tested for antigen reactivity using a cytokine-release assay as described below. Those microcultures exhibiting a significant response of cytokine-release to peptide (at least 2.5-fold over background) were cloned by limiting dilution and expanded in 24-well plates by weekly restimulation with peptides and irradiated autologous PBMCs. Complete culture medium for all procedures consisted of AIM-V medium (Invitrogen/GIBCO, Carlsbad, CA, USA) supplemented with 3% human male AB serum. All blood samples were obtained after the appropriate informed consent.

### Measurement of antigen-specific responses with established CD4 T-cell clones

CD4 T-cells (3 × 10^4^/well) were mixed with irradiated antigen-presenting cells (APCs) in the presence of various concentrations of antigen (peptides, tumor lysates) in 96-well culture plates. APCs consisted of autologous PBMCs (1 × 10^5^/well), HLA-DR expressing L-cells (3 × 10^4 ^well), autologous DCs (5 × 10^3^/well) or LC cell lines (3 × 10^4^/well). The LC cell lines were previously treated with IFN-γ at 500 U/ml for 48 h to enhance HLA-DR expression. The expression of HLA-DR molecules on tumor cells was evaluated by flow cytometry using anti-HLA-DR mAb conjugated with fluorescein isothiocyanate (BD Pharmingen, San Diego, CA). Tumor cell lysates were prepared by 3 freeze-thaw cycles of 1 × 10^8 ^tumor cells, resuspened in 1 ml of serum-free AIM-V medium. Tumor cell lysates served as an antigen at 5 × 10^5 ^cell equivalents/ml. Culture supernatants were collected after 48 h for measuring antigen-induced lymphokine (IFN-γ or GM-CSF) or granzyme B production by the CD4 T-cells using ELISA kits (IFN-γ and GM-CSF; BD Pharmingen, San Diego, CA, USA, granzyme B; Mabtech AB, Nacka Strand, Sweden). To demonstrate antigen-specificity and HLA-DR restriction, blocking of antigen-induced responses were assessed by adding anti-HLA-DR mAb L243 (IgG2a prepared from supernatants of the hybridoma HB-55 obtained from the ATCC) or the anti-HLA-A/B/C mAb W6/32 (IgG2a, ATCC) at 10 μg/ml throughout the 48 h incubation period.

### Cell-mediated cytotoxicity assays

Cytotoxic activity of CD4 T-cell clones was measured using a colorimetric CytoTox 96 assay (Promega, Madison, WI, USA). This system quantifies the release of lactate dehydrogenase (LDH) from target cells. T-cells were mixed with 2 × 10^4 ^target cells at different effector to target (E:T) ratios in 96-round-bottomed-well plates. After 6 h incubation at 37°C, 50 μl supernatant samples were collected from each well to measure LDH content.

### Measurement of peptide-specific responses in LC patients

PBMCs were isolated from fresh heparinized blood by Ficoll-Hypaque centrifugation and washed twice with RPMI 1640. PBMCs were resuspended in complete medium and placed at 1.5 × 10^5 ^cells/well and cultured in triplicate in 96-round-bottomed well plates in the presence of 10 μg/ml peptide. Negative controls (in the absence of peptide), were done in eight replicate samples. One week later, the cultures were restimulated with peptide-pulsed (10 μg/ml) irradiated autologous PBMCs (5 × 10^4^/well). After 7 days of restimulation, supernatants were harvested for examining the ability of peptide-induced lymphokine production by the LC patient's PBMCs. The institutional ethics committee had approved the study protocol and the appropriate informed consent for blood donation was obtained from all patients before blood sampling.

## Results

### Expression of STEAP and EZH2 in LC samples and tumor cell lines

**Figure 1:**
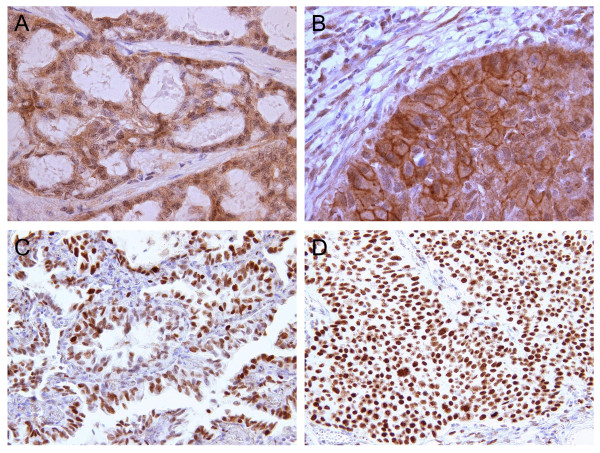
**STEAP and EZH2 expression in LC specimens**. (A) STEAP, adenocarcinoma (×400). (B) STEAP, squamous cell carcinoma (×400). (C) EZH2, adenocarcinoma (×200). (D) EZH2, squamous cell carcinoma (×200).

**Figure 2:**
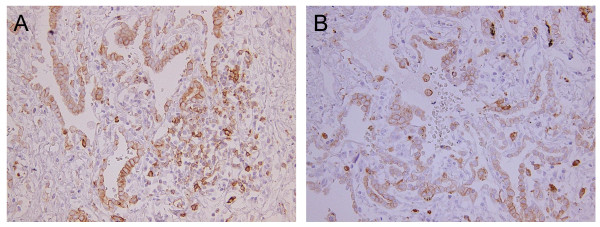
**HLA-DR expression in lung adenocarcinoma specimens**. (A)(B) Adenocarcinoma cells and infiltrating APCs including lymphocytes or alveolar macrophages are positive for HLA-DR (×200).

In immunohistochemistry studies with several surgical specimens of LC, we observed moderate to strong staining with anti-STEAP antibody and anti-EZH2 mAbs. Conversely, staining of adjacent normal tissues with these antibodies was much weaker. STEAP was detected in 8/8 cases of the adenocarcinoma and 6/6 squamous cell carcinoma cases. EZH2 was detected in 5/5 cases of the adenocarcinoma and in 5/5 squamous cell carcinoma cases (examples are shown in Figure 1). In addition, we performed immunohistochemical staining for expression of HLA-DR in 5 lung adenocarcinomas and 4 lung squamous cell carcinomas. We have found the expression of HLA-DR in the adenocarcinoma cells (4/5 cases) and in the infiltrating APCs, lymphocytes and alveolar macrophages (Figure 2). These observations suggest that tumor cells could be logical targets of STEAP- or EZH2-specific CD4 T-cells *in situ*.

**Figure 3:**
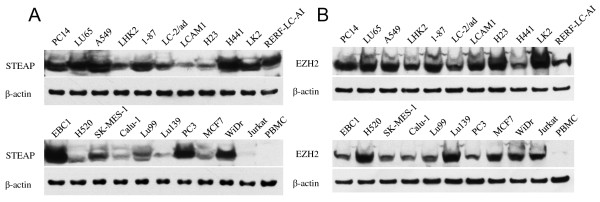
**Expression of STEAP and EZH2 protein in various tumor cell lines**. Western blotting was done using STEAP- or EZH2-specific antibody as described in "Materials and methods". The STEAP protein has a mass of approximately 36 kDa, and EZH2 protein 91 kDa. (A) STEAP, (B) EZH2.

Western blot analysis revealed that STEAP was present in several tumor cell lines including LCs (except for Jurkat T cell lymphoma, which served as a negative control), but was absent in PBMC samples from healthy individuals (Figure 3A). EZH2 was detected in all the tumor cell lines (including Jurkat), but not in PBMCs (Figure 3B). These observations provide a rationale for exploring the use of STEAP and EZH2 as TAAs for developing anti-tumor T-cell based immunotherapy for LC (and the other tumor types).

### Selection of potential HLA-class II restricted peptide epitopes from EZH2 and STEAP

To identify the promiscuous (broadly degenerate) HLA-class II binding peptide epitopes is very important since using the degenerate epitope-based vaccines in applicable to general population. For predicting promiscuous HLA-class II binding peptides, we used computer-based MHC peptide binding motifs for three common HLA-class II molecules, HLA-DR1, DR4 and DR7 [14]. In addition, this algorithm indicate that some peptides that score high for the DR1, DR4 and DR7 alleles also have the capacity to bind to additional HLA-class II alleles such as DR9, DR13, DR15 and DR53. Therefore, we have reported that some peptides predicted by additional HLA-class II alleles, such as DR9, DR13, DR15 or DR53 [17]. In this study, the prediction system could select nine peptide sequences from EZH2 (data not shown) and we selected the three highest potentially promiscuous peptides, EZH2_95-109_, EZH2_220-234 _and EZH2_693-704_. In the case of STEAP, the algorithm system suggested 28 peptide sequences (data not shown) as potentially promiscuous HLA-class II binders and we have reported that two STEAP peptides, STEAP_102-116 _and STEAP_192-206 _were effective in stimulating *in vitro *anti-tumor T helper responses [18,19]. In the present study, considering that an ideal TAA should contain peptide epitopes to stimulate both CD8 and CD4 T-cells, we selected 2 STEAP peptides from these promiscuous binders, STEAP_261-275_, which overlaps with HLA-A2-restricted CD8 T-cell epitope STEAP_262-270 _[20] and STEAP_281-296_, which lies proximal to HLA-A2-restricted CD8 epitope, STEAP_292-300 _[21]. We synthesized the three EZH2 and two STEAP epitope peptides and proceeded to determine whether these peptides could induce CD4 helper T-cell responses *in vitro*.

### Induction of CD4 T-cell responses to STEAP and EZH2 peptide epitopes

**Figure 4:**
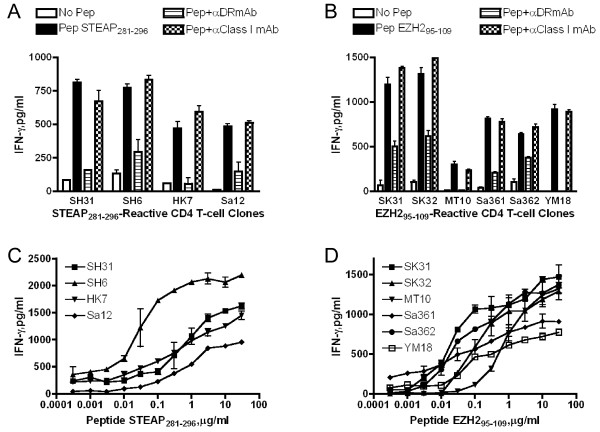
**HLA-DR restriction analysis of STEAP_281-296_- and EZH2_95-109_-reactive CD4 T-cell clones**. (A) L243 but not W6/32 inhibited IFN-γ production from HLA-DR restricted, STEAP_281-296_-reactive CD4 T-cell clones. (B) HLA-DR restriction analysis of EZH2_95-109_-reactive CD4 T-cell clones. (C and D) STEAP_281-296_- and EZH2_95-109_-reactive CD4 T-cell clones were tested for their capacity to recognize autologous PBMCs as APCs in the presence of various concentrations of peptide. Points means of triplicate determinations, bars SD. Points without bars had SD < 10% the value of the mean. Results are representative of two experiments that were done with the same samples.

**Figure 5:**
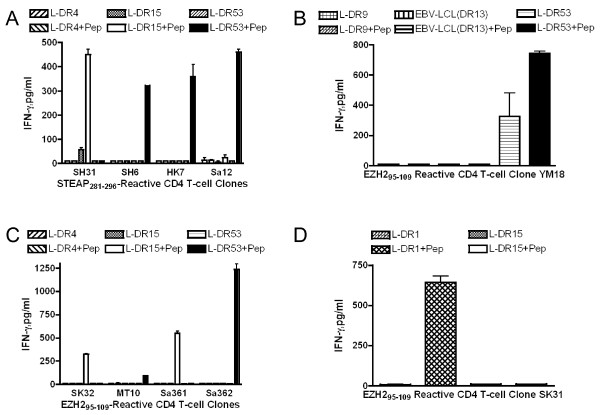
**Definition of restricting HLA-DR alleles of the STEAP- or EZH2-reactive CD4 T-cell clones**. IFN-γ production of the STEAP_281-296_-reactive CD4 T-cell clones (A) and EZH2_95-109_-reactive CD4 T-cell clones (B, C, and D) was evaluated using L-cells or semi-allogenic EBV-LCLs homozygous for HLA-DR molecules as APCs to define the restricting HLA-DR elements. Columns means of triplicate determinations, bars SD. Columns without bars had SD < 10% the values of the mean. Results are representative of at least two experiments.

Next, we evaluated whether these peptides were capable of stimulating CD4 T-cells obtained from 6 healthy individuals (HLA-DR4/15, HLA-DR4/9, HLA-DR4/15, HLA-DR1/15, HLA-DR9/13 and HLA-DR9/14) using autologous DCs as APCs. After 2-3 peptide restimulation cycles, 2 of 5 peptides (STEAP_281-296 _and EZH2_95-109_) were able to induce peptide-specific CD4 T-cell responses. To carry out more detailed antigen specificity and HLA-DR restriction analyses, and to assess tumor cell recognition, T-cell clones (4 reactive with STEAP_281-296 _and 6 reactive with EZH2_95-109_) were isolated by limiting dilution. As shown in Figure 4, all of these clones recognized their respective peptide epitopes in a dose dependent manner and this recognition was inhibited by anti-HLA-DR (L243) mAb but not by anti-HLA-class I (W6/32) mAb. Since mAb L243 is specific for HLA-DR, these results indicate that the presentation of STEAP_281-296 _and EZH2_95-109 _to the T-cells is via HLA-DR molecules and not via other HLA-class II molecules (HLA-DQ or DP). To more specifically delineate the HLA-DR restriction alleles, we studied the T-cells responses using a panel of HLA-DR transfected mouse L-cells and HLA-typed human EBV-LCLs. As shown in Figure 5A, the STEAP_281-296_-reactive clone SH31 was restricted by HLA-DR15 molecules, and clones SH6, HK7 and Sa12 recognized the same peptide but in the context of HLA-DR53. The EZH2_95-109 _epitope was presented to clone SK31 via HLA-DR1, to clones SK32 and Sa361 by HLA-DR15 and to clones MT10, Sa362 and YM18 by HLA-DR53 (Figure 5B, C and 5D). These results indicate that STEAP_281-296 _and EZH2_95-109 _can be presented to CD4 T-cells by more than one HLA-DR allele, behaving as classical promiscuous epitopes.

### Direct recognition of STEAP- or EZH2-expressing LC cells by STEAP_281-296_- or EZH2_95-109_-reactive CD4 T-cell clones

**Figure 6:**
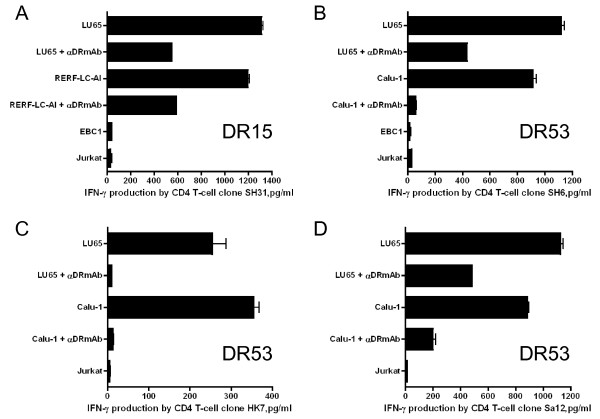
**Direct recognition of STEAP-expressing lung tumor cells by STEAP_281-**296**_**-**reactive CD4 T-cell clones**. DR15-restricted CD4 T-cell clone SH31 (A) and DR53-restricted CD4 T-cell clones SH6 (B), HK7 (C), and Sa12 (D) were tested for their capacity to recognize antigen directly on STEAP positive LC cells that are either matched or mismatched for the restricting HLA-DR alleles. The HLA-DR-negative Jurkat was also used as negative control. The antigen specificity of these responses was demonstrated by blocking tumor recognition with L243. Columns without bars had SD < 10% the values of the mean. Results are representative of two separate experiments.

**Figure 7:**
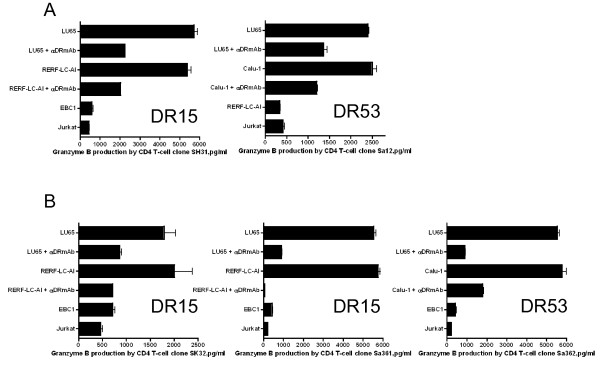
**Granzyme B production from STEAP_281-296_- or EZH2_95-109_-reactive CD4 T-cell clones**. STEAP_281-296_-reactive, DR15-restricted CD4 T-cell clone SH31 (A, left) and DR53-restricted CD4 T-cell clone Sa12 (A, right) were able to secrete granzyme B reacting with STEAP+/HLA-DR matched LC cells. EZH2_95-109_-reactive, DR15-restricted CD4 T-cell clone SK32 (B, left) and Sa361 (B, middle), and DR53-restricted CD4 T-cell clone Sa362 (B, right) were able to secrete granzyme B reacting with EZH2+/HLA-DR matched LC cells. The HLA-DR-negative Jurkat was also used as negative controls. The antigen specificity of these responses was demonstrated by blocking tumor recognition with L243. Columns without bars had SD < 10% the values of the mean. Results are representative of two separate experiments.

**Figure 8:**
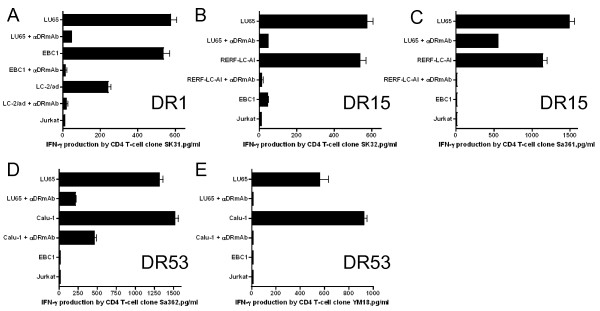
**Direct recognition of EZH2-expressing lung tumor cells by EZH2_95-109_-reactive CD4 T-cell clones**. DR1-restricted CD4 T-cell clone SK31 (A), DR15-restricted CD4 T-cell clone SK32 (B), Sa362 (C), DR53-restricted CD4 T-cell clones Sa362 (D) and YM18 (E) were tested for their capacity to recognize antigen directly on EZH2 positive LC cells that are either matched or mismatched for the restricting HLA-DR alleles. The HLA-DR-negative Jurkat was also used as negative controls. The antigen specificity of these responses was demonstrated by blocking tumor recognition with L243. Columns without bars had SD < 10% the values of the mean. Results are representative of two separate experiments.

We then evaluated whether STEAP or EZH2 reactive CD4 T-cell clones could directly recognize STEAP or EZH2 expressing LC cells. Before this study, we first measured cell surface HLA-DR expression in several tumor cell lines that would be used in these studies. The LC cell lines (LU65, LC-2/ad, RERF-LC-AI, EBC1, Calu-1) displayed measurable levels of surface HLA-DR after IFN-γ treatment. However, there was no HLA-DR expression in A549 lung adenocarcinoma and Jurkat T-cell lymphoma (data not shown). The peptide-reactive CD4 T-cell clones were then tested for reactivity (IFN-γ production) against the HLA-DR expressing, STEAP/EZH2 positive LC cell lines. As shown in Figure 6A, CD4 T-cell clone SH31 (STEAP_281-296_-reactive, DR15-restricted) recognized two STEAP+/DR15+ LC tumors (LU65, RERF-LC-AI). Similarly, the HLA-DR53-restricted, STEAP_281-296_-reactive CD4 T-cell clones, SH6, HK7 and Sa12 recognized STEAP+/DR53+ LC tumors (LU65, Calu-1, Figure 6B, C and 6D). In addition, the STEAP_281-296_-reactive CD4 T-cell clones SH31 and Sa12 produced granzyme B (serine protease cytotoxic molecule) when stimulated with LC tumors in an HLA-DR dependent manner (Figure 7A). In the case of EZH2-reactive CD4 T-cell responses, clone SK31 (EZH2_95-109_-reactive, DR1-restricted) recognized several EZH2+/DR1+ LC tumors (LU65, EBC1 and LC-2/ad, Figure 8A). CD4 T-cell clones SK32 and Sa361 (EZH2_95-109_-reactive, DR15-restricted) recognized EZH2+/DR15+ LC tumors (LU65, RERF-LC-AI, Figure 8B and 8C). Lastly, as shown in Figure 8D and 8E, CD4 T-cell clones Sa362 and YM18 (EZH2_95-109_-reactive, DR53-restricted) were able to react with EZH2+/DR53+ LC tumors (LU65, Calu-1). Furthermore, the EZH2_95-109_-reactive CD4 T-cell clones produced granzyme B when stimulate with LC cells (Figure 7B). Direct tumor recognition in all cases was antigen-specific and HLA-DR-restricted since tumor cell lines not expressing the appropriate antigen (STEAP, EZH2), and the corresponding HLA-DR molecule failed to stimulate the CD4 T-cell responses. Furthermore, the capacity of the STEAP- and EZH2-expressing LC cells to activate the CD4 T-cell clones was significantly inhibited by the addition of anti-HLA-DR L243 mAb confirming that the peptide epitopes were presented via HLA-DR molecules.

### Recognition of naturally processed exogenous antigen by STEAP-reactive CD4 T-cell clones

**Figure 9:**
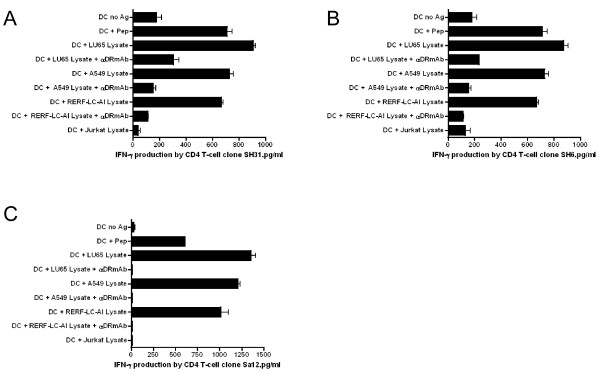
**STEAP_281-296_-reactive CD4 T-cell recognize naturally processed exogenous antigen presented by autologous DCs**. STEAP_281-296_-reactive CD4 T-cell clones SH31 (A), SH6 (B), and Sa12 (C) were tested for their reactivity against STEAP positive LC cell (LU65, A549, and RERF-LC-AI) lysates and STEAP negative Jurkat cell lysate when presented by autologous DCs. The capacity of L243 to inhibit these responses was assessed to demonstrate that T-cell reactivity was through TCR/HLA interactions. Columns means of triplicate determinations, bars SD. Columns without bars had SD < 10% the values of the mean. Results are representative of at two separate experiments.

In addition, we assessed whether APCs such as DCs could capture and process antigens derived from dead STEAP expressing tumor cells (freeze-thaw cell lysates) and present the STEAP epitope to the CD4 T-cell clones. As shown in Figure 9, the STEAP_281-296_-reactive CD4 T-cell clones SH31, SH6 and Sa12 recognized naturally processed epitope presented by DCs derived from STEAP positive LC cell lysates (LU65, A549, RERF-LC-AI) but not STEAP negative, Jurkat cell lysates. These responses were inhibited by L243 mAb (anti-HLA-DR) but not by W6/32 mAb (anti-HLA-class I), indicating that CD4 T-cell responses were antigen-specific and via the interaction of T-cell receptor with HLA-DR molecules. These results indicate that the STEAP_281-296 _epitope can be presented to T-cells via either the endogenous (direct tumor recognition) or exogenous (DCs) antigen processing pathways.

Conversely, the EZH2-reactive CD4 T-cell clones were unable to recognize naturally processed epitopes derived from tumor cell lysates via DCs (data not shown).

### Cytotoxic activity of STEAP- or EZH2-specific CD4 T-cell clones

**Figure 10:**
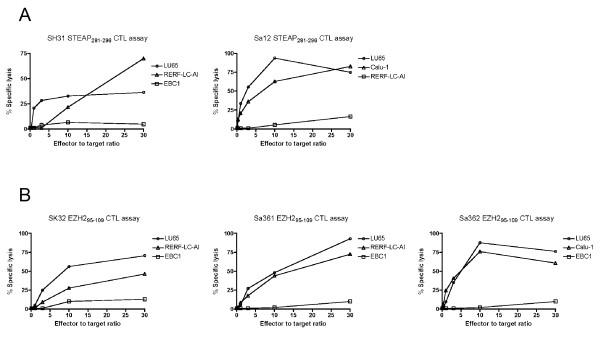
**Assessment of cytolytic activity of STEAP_281-296_- or EZH2_95-109_-reactive CD4 T-cell clones**. The DR15-restricted, STEAP_281-296_-reactive CD4 T-cell clone SH31 (A, left), and DR53-restricted, STEAP_281-296_-reactive CD4 T-cell clone Sa12 (A, right) were evaluated for their capacity to kill STEAP-expressing, DR53 positive LC cells (LU65 and Calu-1) or STEAP-expressing, DR53 negative LC cells (EBC1, negative control). The DR15-restricted, EZH2_95-109_-reactive CD4 T-cell clones SK32 (B, left), Sa361 (B, middle), and DR53-restricted, EZH2_95-109_-reactive CD4 T-cell clone Sa362 (B, right) were evaluated for their capacity to kill EZH2-expressing, DR15 positive LC cells (LU65 and RERF-LC-AI), DR53 positive LC cells (LU65 and Calu-1) or EZH2-expressing, DR15 and DR53 negative LC cells (EBC1, negative control). Points means of triplicate determinations, bars SD. Points without bars had SD < 10% the value of the mean.

Having observed that peptide STEAP_281-296 _and EZH2_95-109 _elicited CD4 T-cell clones produced granzyme B when stimulated with STEAP or EZH2 expressing LC cells (Figure 7), we evaluated the cytotoxic activity of the CD4 T-cell clones. The STEAP_281-296_-reactive, DR15-restricted clones SH31 and Sa12 effectively lysed STEAP expressing LC cell lines in a dose dependent manner (Figure 10A). Similarly, the EZH2_95-109_-reactive clones SK32 and Sa361 and Sa362 were efficient in killing the EZH2 expressing LC cell lines (Figure 10B).

### STEAP and EZH2 peptide recognition by PBMCs from patients with LC

Lastly, to further evaluate the capacity of LC patients to exhibit CD4 T-cell responses to STEAP_281-296 _and EZH2_95-109_, we stimulated PBMCs from 10 LC patients and 3 healthy donors with the corresponding peptides in short-term cultures. Seven days after the second stimulation, culture supernatants were collected and IFN-γ or GM-CSF production was assessed. Peptide TT_830-843 _was used as a positive control since this antigen has ability to induce strong CD4 T-cell responses in the majority individuals regardless of their HLA-DR alleles. The results presented in Table 1 show significant T-cell responses to STEAP_281-296 _in 9/10 patients, to EZH2_95-109 _in all patients, and to the positive control TT_830-843 _in all patients. These results suggest that T-cell precursors that are reactive with the STEAP and EZH2 epitopes exist in the peripheral blood of patients with LC.

**Table 1:** Assessment of T-cell responses to the STEAP_281-296 _and EZH2_95-109_-peptides in lung cancer patients and healthy donors.

No.	Sex	Age (y)	Pathological stage	Histological diagnosis	No antigen (IFN-γ, pg/ml)	No antigen (GM-CSF, pg/ml)	STEAP_281-296 _(IFN-γ, pg/ml)	STEAP_281-296 _(GM-CSF, pg/ml)	EZH2_95-109 _(IFN-γ, pg/ml)	EZH2_95-109 _(GM-CSF, pg/ml)	TT_830-843 _(IFN-γ, pg/ml)	TT_830-843 _(GM-CSF, pg/ml)
1	F	61	M1a IV	adenocarcinoma	<	<	259.7 ± 74.6	749.4 ± 196.9	220.8 ± 175.3	968.5 ± 159.7	95.9 ± 9.2	545.7 ± 25.3
2	M	80	T3N0M0 IIB	adenosquamous carcinoma	<	<	320.5 ± 267.2	893.0 ± 355.6	565.7 ± 38.0	997.3 ± 27.4	985.1 ± 799.0	1267.8 ± 260.9
3	F	71	T1aN0M0 IA	adenocarcinoma	41.0 ± 5.0	35.8 ± 2.5	704.3 ± 502.5	516.0 ± 87.1	1030.0 ± 106.6	147.4 ± 55.7	1004.4 ± 451.3	620.8 ± 472.2
4	M	81	T3N0M0 IIB	adenocarcinoma	<	<	516.8 ± 0	206.0 ± 93.9	94.9 ± 0	65.9 ± 32.3	57.1 ± 25.1	77.9 ± 12.7
5	M	74	T1bN0M0 IA	adenocarcinoma	<	<	31.1 ± 0	135.4 ± 0	31.1 ± 20.1	34.8 ± 0	64.2 ± 0	31.2 ± 0
6	F	69	T3N0M0 IIB	adenocarcinoma	<	<	487.6 ± 444.3	474.9 ± 188.5	473.1 ± 354.0	389.3 ± 70.1	550.3 ± 118.1	917.4 ± 147.4
7	M	74	T2aN0M0 IB	basaloid carcinoma	<	<	189.7 ± 76.9	<	152.7 ± 57.5	<	203.9 ± 0	299.7 ± 278.1
8	M	71	T1aN0M0 IA	adenocarcinoma	<	<	173.7 ± 7.3	105.2 ± 49.9	54.3 ± 2.7	158.4 ± 56.7	91.4 ± 62.1	180.8 ± 1.7
9	F	56	T2aN0M0 IB	adenocarcinoma	<	<	<	<	134.7 ± 0	<	91.9 ± 0	<
10	F	60	T1aN1M0 IIA	adenocarcinoma	<	<	<	43.0 ± 38.3	44.2 ± 5.0	287.9 ± 337.1	234.3 ± 91.3	172.3 ± 58.3
11	M	32	-	-	<	<	<	440.6 ± 110.0	<	598.9 ± 105.1	<	301.8 ± 52.2
12	M	44	-	-	<	<	214.3 ± 50.6	122.5 ± 20.9	148.2 ± 18.1	<	492.7 ± 312.7	156.7 ± 39.0
13	M	38	-	-	<	<	180.1 ± 45.5	319.6 ± 64.7	190.4 ± 40.2	334.2 ± 57.1	246.8 ± 81.0	414.4 ± 115.1

<: less than the lower limit of detection (mean < 30 pg/ml). No. 1 to 10 are lung cancer patients and No. 11 to 13 are healthy donors. IL-4, Il-5, IL-17, and IL-10 are not detected in all patients and healthy donors.

IFN-γ and GM-CSF productions of all above cases were inhibited by anti-HLA-DR mAb L243 (mean < 30 pg/ml).

## Discussion

T-cell based immunotherapy has become an accepted treatment alternative for cancer. The presence of lymphocytic infiltrations in murine and human lung tumors suggests that an immune reaction could potentially help to control LC progression [22,23]. In view of this, we believe that identifying TAAs and the corresponding peptide epitopes capable of eliciting anti-tumor T-cell responses will be critical to develop peptide-based cancer immunotherapy. EZH2 plays a role in cell cycle regulation and proliferation and increased EZH2 expression has been correlated with aggressive tumor behavior and poor survival in LC [18]. STEAP is overexpressed in various types of tumors including LC. In addition, it has been reported that generation of CD4 or CD8 T lymphocytes recognizing STEAP and EZH2 indicates that STEAP and EZH2 could be potent TAAs for T-cell based immunotherapy [18-21,24].

The goal of the present study was to assess whether STEAP and EZH2 could function as TAAs for LC, capable of generating anti-tumor CD4 T-cell responses. The results showed that two peptides, STEAP_281-296 _and EZH2_95-109_, were efficient in activating and expanding CD4 T-cells obtained from healthy individuals and LC patients. The results show that STEAP_281-296 _could be presented to CD4 T-cells by either HLA-DR15 or DR53 and that EZH2_95-109 _is presented in the context of DR1, DR15 or DR53 (Figure 5). The HLA-DR53 molecule is expressed by a large proportion of the population (~50%) owing to its linked co-expression with the HLA-DR4, DR7, and HLA-DR9 alleles [14]. Thus, each peptide would provide broad coverage for clinical use. Most significant was the demonstration that the CD4 T-cell clones generated by STEAP_281-296 _and EZH2_95-109 _were capable of recognizing naturally processed antigen directly on HLA-DR expressing tumor cells and that the T-cells exhibited cytotoxicity against the tumor cells. These findings suggest the possibility of direct therapeutic benefit of CD4 anti-tumor T-cell responses independent of CD8 T-cells. Both STEAP_281-296 _and EZH2_95-109 _epitope peptides were able to stimulate T-cell responses in LC patients indicating that T-cell precursors capable of recognizing STEAP_281-296 _or EZH2_95-109 _exist in both healthy individuals and some patients with LC.

As with the many TAA such as CEA, PSA or Her2/neu, message and protein levels of EZH2 or STEAP appear to be low amounts in normal tissues. Our results show that normal cells such as PBMCs and DCs were not recognized by the STEAP or EZH2-reactive CD4 T-cells. Furthermore, Western blot analysis revealed no detectable STEAP or EZH2 expression in PBMCs and no significant expression of STEAP or EZH2 was found in normal tissues using immunohistochemical staining. Thus, we believe that no significant autoimmune pathology would be elicited if STEAP or EZH2 CD4 T-cell epitopes were used for cancer immunotherapy. Nevertheless, it would be wise to assess in more detail the levels of STEAP or EZH2 in other normal tissues (*e.g.*, liver, kidney, brain) before setting about clinical trials using STEAP or EZH2 as targets for immunotherapy.

It is possible that direct stimulation of T-cells, even priming by tumor cells may be actually tolerant toward TAAs. In fact, EZH2 and STEAP express in very low amounts in normal tissues and benign neoplasms and upregulated and overexpressed in malignant tumors. These low- and high-level expressions may induce tolerance by deletion of the TAA-specific T-cell repertoire, especially T-cells with high affinity for its peptide epitope. However, it appears that tolerance induction by the tumors expressing, STEAP or EZH2 may not be leaded, because we observed that cancer patient's PBMC could respond to the peptides and produced lymphokines. These results suggest that the STEAP- or EZH2-reactive T-cells are not tolerant and completely deleted by tumor cells *in vivo*.

The present study is the first report of the identification of an immunogenic epitope of EZH2 recognized by CD4 T-cell and suggests that EZH2, which is the overexpressed TAA in LC, could be significant immunogenic target for inducing both CD4 and CD8 T-cell against LC. In addition, we present new CD4 T-cell epitope STEAP_281-296_, which is closely located in the HLA-A2 restricted CD8 T-cell epitope, STEAP_292-300 _[21]. Wroblewski et al demonstrated that some LC cell lines (23%) constitutively express HLA-DR molecules and treatment of IFN-γ could enhance expression of HLA-DR molecules in LC cells [25]. Another published studies described that 45% of lung adenocarcinoma specimens expressed HLA-DR [26] and HLA-DR molecules on primary tumors were detected in 10% of patients with micrometastasis to regional lymph nodes and in 30% of patients without tumor spread [27]. We were also able to verify that the expression of HLA-DR was found in some lung adenocarcinomas and tumor adjacent infiltrating APCs (Figure 2). These observations suggest that CD4 T-cells have important role in immunological response to LC and tumor cells are logical targets of the TAA-specific CD4 T-cells *in vivo*. In addition, the predicted peptide STEAP_281-296 _and EZH2_95-109 _could be capable of stimulating T-cell responses in some LC patients. Taken together these results, we believe that the simultaneous induction of STEAP- and EZH2-specific CD4 and CD8 T-cell responses would be more effective in achieving anti-tumor effects than the separate induction of CD4, or CD8 T-cell responses. Moreover, the recent development of EZH2 inhibitors as anti-cancer therapeutics [9] opens the interesting opportunity of a combined chemotherapy/immunological approach for treating EZH2-expressing LCs.

## Conclusions

Peptides STEAP_281-296 _and EZH2_95-109 _are newly described CD4 T-cell epitopes that can elicit effective anti-tumor T-cell responses against STEAP or EZH2 expressing LC cell lines. These peptides may facilitate the development of peptide-based vaccines for LC.

## Abbreviations

LC: lung cancer; NSCLC: non-small cell lung cancer; TAA: tumor-associated antigen; STEAP: six-transmembrane epithelial antigen of the prostate; EZH2: enhancer of zeste homolog 2; L-cell: mouse fibroblast cell line; PBS: phosphate buffered saline; mAb: monoclonal antibody; HPLC: high-performance liquid chromatography; TT: tetanus toxoid; PBMC: peripheral blood mononuclear cell; DC: dendritic cell; APC: antigen-presenting cell; LDH: lactate dehydrogenase; E:T: effector to target.

## Competing interests

The authors declare that they have no competing interests.

## Authors' contributions

SH carried out and participated in all of the studies. TK, YM, NA, KS, SK, MT have made substantial contributions to acquisition of data. MK provided the clinical samples. EC and HK designed, supervised and coordinated the study, and drafted the manuscript. All authors read and approved the final manuscript.
